# EML4-ALK-mediated activation of the JAK2-STAT pathway is critical for non-small cell lung cancer transformation

**DOI:** 10.1186/s12890-021-01553-z

**Published:** 2021-06-06

**Authors:** Ying Li, Yongwen Li, Hongbing Zhang, Ruifeng Shi, Zihe Zhang, Hongyu Liu, Jun Chen

**Affiliations:** 1 School of Chemical Engineering and Technology, Tianjin University, Tianjin, 300072 China; 2 Department of Lung Cancer Surgery, Laboratory of Lung Cancer Metastasis and Tumor Microenvironment, Tianjin Lung Cancer Institute, Tianjin Medical University General Hospital, Tianjin, 300052 China

**Keywords:** EML4-ALK, JAK2-STAT pathway, Non-small cell lung cancer, Transformation

## Abstract

**Background:**

The echinoderm microtubule-associated protein-like-4 anaplastic lymphoma kinase (EML4-ALK) fusion gene was identified in a subset of non-small cell lung cancer (NSCLC) patients. They responded positively to ALK inhibitors. This study aimed to characterize the mechanisms triggered by EML4-ALK to induce NSCLC transformation.

**Methods:**

HEK293 and NIH3T3 cells were transfected with EML4-ALK variant 3 or pcDNA3.1-NC. H2228 cells were transfected with siRNA-EML4-ALK or siRNA-NC. Cell viability and proliferation were measured by the CCK-8 and EdU methods, respectively. Flow cytometry revealed apoptosis. Gene expression profiles were generated from a signaling pathway screen in EML4-ALK-regulated lung cancer cells and verified by qPCR and Western blotting. The co-immunoprecipitation and immunohistochemistry/ immunofluorescence determined the interaction and colocalization of JAK2-STAT pathway components with EML4-ALK.

**Results:**

Microarray identified several genes involved in the JAK2-STAT pathway. JAK2 and STAT6 were constitutively phosphorylated in H2228 cells. EML4-ALK silencing downregulated phosphorylation of STAT6. Expression of EML4-ALK in HEK293 and NIH3T3 cells activated JAK2, STAT1, STAT3, STAT5, and STAT6. In EML4-ALK-transfected HEK293 cells and EML4-ALK-positive H2228 cells, activated STAT6 and JAK2 colocalized with ALK. STAT3 and STAT6 were phosphorylated and translocated to the nucleus of H2228 cells following IL4 or IL6 treatment. Apoptosis increased, while cell proliferation and DNA replication decreased in H2228 cells following EML4-ALK knockdown. In contrast, HEK293 cell viability increased following EML4-ALK overexpression, while H2228 cell viability significantly decreased after treatment with ALK or JAK-STAT pathway inhibitors.

**Conclusions:**

Our data suggest that the aberrant expression of EML4-ALK leads to JAK2-STAT signaling pathway activation, which is essential for the development of non-small cell lung cancer.

## Background

Non-small cell lung cancer (NSCLC) is the most malignant tumor in the world [1]. The prognosis for patients with NSCLC has improved over the last years, mainly thanks to targeted therapy. In contrast to traditional chemotherapy and radiotherapy, targeted cancer therapy has gained increasing attention for its greater tumor specificity, higher efficacy, and decreased toxicity [2]. Indeed, targeted therapy prolonged the survival of NSCLC patients with a specific genotype. For example, patients with NSCLC who harbored epidermal growth factor receptor (EGFR) mutations benefited from EGFR tyrosine kinase inhibitors (TKIs), such as gefitinib and erlotinib [3, 4].

The fusion gene echinoderm microtubule-associated protein-like 4 (EML4)-anaplastic lymphoma kinase (ALK) is present in approximately 5% of NSCLC patients. It results from a recurrent chromosome inversion [5]. The EML4-ALK fusion appears to be more common in female and non-smoking patients with adenocarcinoma [6]. It does usually occur in NSCLC without EGFR mutation. Patients who harbor EML4-ALK fusions seem not responsive to EGFR TKIs or traditional chemotherapy but are sensitive to ALK inhibitors [6]. Various break and fusion points within the EML4 locus in NSCLC cells give rise to different isoforms of EML4-ALK [5–11]. The most common EML4-ALK variants are variants 1 and 3, which account for about 60% of EML4-ALK-positive lung cancer cases.

Mouse NIH3T3 cells expressing human EML4-ALK fusion formed foci in culture and subcutaneous tumors in nude mice, indicating that EML4-ALK might be a key factor for the development of lung cancer [12]. Inhibitors specific for ALK reportedly suppress the growth and induce apoptosis of lung cancer cells [13–15]. Therefore, ALK inhibition might constitute a potential strategy for the treatment of EML4-ALK-positive NSCLC. However, the molecular mechanisms involved in regulating cell proliferation and survival of lung cancer cells by EML4-ALK are still unknown.

This study found that EML4-ALK activated the Janus kinase 2-signal transducers and activators of the transcription (JAK2-STAT) signaling pathway. We also identified changes in the expression of multiple STAT target genes using high-throughput microarray analysis. We hypothesized that the JAK2-STAT pathway plays an important role in developing lung cancer driven by EML4-ALK. EML4-ALK fusion gene phosphorylated JAK2, and constitutively activated STAT-1, STAT-3, STAT-5, and STAT-6 resulting in the increased viability of EML4-ALK-positive cells.

## Methods

### Cell culture, cytokines, and inhibitors

Human embryo kidney (HEK293) cells do not express STAT6 and the human lung adenocarcinoma cell line H2228, expressing STAT6 and harboring the EML4-ALK variant 3 fusion gene, were obtained from the American Type Culture Collection. The NIH3T3 mouse embryonic fibroblasts were obtained from the Institute of Biochemistry and Cell Biology, the Chinese Academy of Sciences (Shanghai, China). All cell lines were cultured in Gibco™ Dulbecco’s modified Eagle medium supplemented with 10% FBS and 1% penicillin–streptomycin at 37 °C with 5% CO_2_. IL4 and IL6 were purchased from PeproTech (USA), diluted in sterile ultrapure water as indicated by the manufacturer, and frozen in aliquots at − 80 °C. The small molecule inhibitors TAE684 and SH-4-54 were purchased from Selleck Chemicals (USA), diluted, and stored according to the manufacturer’s instructions.

### Patients and samples

EML4-ALK-positive samples were obtained from seven NSCLC patients, as previously described [10]. EML4-ALK-negative patients constituted the control group. Each patient provided written informed consent, and the Ethics Committee of Tianjin Medical University General Hospital approved the study. Table 1 lists the demographic and clinical characteristics of the EML4-ALK-positive patients included in this study.

**Table 1:** Characteristics of patients with EML4-ALK-positive NSCLC and the associated JAK2 and STAT6 protein expression

Case number	Smoking status	Histology (H&E)	pTNM	ALK/P-JAK2/P-STAT6 IHC at primary sites	ALK/P-JAK2/P-STAT6-IHC at metastatic sites
1-147	Non	AD	T2aN2M0	+ / + / +	–/–/–
2-159	Non	Mixed-AD	T1N2M0	+ / ±	+ / ±
3-161	Non	Mixed-AD	T1N2M0	+ / + / +	–/–/–
4-170	Non	Ad + SCC	T2bN2M0	+ / ±	–/–/–
5-177	Non	Mixed -AD	T2aN1M0	+ / + / +	+ / ±
6-184	Yes	Mixed -AD	T2aN1M1	+ / + / +	+ / ±
7-98	Non	Mucinous BAC	T2aN0M0	+ / + / +	/

*AD* adenocarcinoma, *SCC* squamous carcinoma, *BAC* bronchioloalveolar carcinoma, + positive, − negative

### Microarray gene expression analysis

Biotinylated target cRNA was prepared from total RNA isolated from H2228 cells using the Affymetrix One-cycle cDNA synthesis kit following the manufacturer’s instructions (Affymetrix, Santa Clara, CA). Then the biotinylated cRNA was fragmented and hybridized to the GeneChip Human Genome U133 plus 2.0 array (Affymetrix, Inc.), which contains more than 54,000 transcripts and expressed sequence tags. Raw data were analyzed with the Affymetrix GeneChip Operating Software (GCOS) 1.4 and filtered using twofold expression levels. The *P*-value cutoff was 0.05. The annotations were analyzed using a combination of interactive NetAffx query (www.affymetrix.com) and R suite. Clustering analysis was performed using MultiExperiment Viewer ( The Institute for Genomic Research, http://www.tigr.org/tdb/microarray/).

### Immunohistochemistry and immunofluorescence

Immunohistochemistry and immunofluorescence were performed as previously described [10]. Sections were washed in PBS and blocked with 5% bovine serum albumin for 15 min at room temperature. For immunohistochemistry, the sections were then incubated with the primary antibody against ALK (1:50, DAKO North America, USA), phosphorylated JAK2 (p-JAK2), phosphorylated STAT3 (P-STAT3), or phosphorylated STAT6 (p-STAT6) (1:50, Cell Signaling Technology, Inc., USA) at 4 °C overnight. In negative controls, PBS replaced the antibody. Cytoplasmic staining was considered positive for ALK.

For immunofluorescence, the anti-ALK antibodies (DAKO North America, USA) were used at a dilution of 1:50, and the antibodies against p-JAK2, p-STAT3, and p-STAT6 antibodies (Cell Signaling Technology, Inc., USA) at a dilution of 1:100 for 2 h at 37 °C. AlexaFluor 488-conjugated goat anti-rabbit (Invitrogen) and Alexa Fluor 594-conjugated goat anti-mouse were used as secondary antibodies. Images were taken with an inverted fluorescence microscope (NIKON, Tokyo, Japan).

### Generation of siRNAs and plasmids

The EML4-ALK siRNA oligonucleotides (target sequence: CCTGTCAGCTCTTGAGTCA, sense strand: 5ʹ-CCUGUCAGCUCUUGAGUCA-dTdT-3ʹ, antisense strand: 5ʹ-dTdT-GGACAGUCGAGAACUCAGU-3ʹ) were synthesized by RiboBio (Guangzhou, China). A scrambled siRNA duplex was used as a negative control (siRNA-NC). The EML4-ALK variant 3 cDNA expression construct was engineered by cloning the EML4-ALK PCR product generated from H2228 cells into the pcDNA3.1 vector (Invitrogen) using EcoRI and XbaI sites (forward: 5ʹ-CGGAATT CACTCTGTCGGTCCGCTGAATGAA-3ʹ and reverse: 5ʹ-GCTCTAGACCAC GGTCTTAGGGATCCCAAGGAAGAGAA-3ʹ).

### Protein extraction, immunoprecipitation, and Western blotting

For the preparation of total cell lysates, cells were washed with PBS and lysed on ice for 30 min in lysis buffer (50 mM Tris–HCl, pH 7.5, 150 mM NaCl, 2 mM EDTA, 1% Triton-X100) containing a protease inhibitor cocktail (Roche, Mannheim, Germany), 50 mM NaF, and 1 mM Na_3_VO4. The samples were centrifuged at 12,000 rpm for 15 min at 4 °C, and the proteins quantified using the BCA protein assay (Thermo Scientific, MA, USA) with bovine serum albumin as standard. Equal amounts of proteins (10–40 μg/lane) were separated by sodium dodecyl sulfate–polyacrylamide gel electrophoresis (SDS-PAGE), and transferred to nitrocellulose membranes (Amersham Biosciences, NJ). After washing, membranes were incubated with the primary antibody (1:1000 dilution) overnight at 4 °C under gentle rocking. Primary antibodies against ALK, JAK2, STAT1, STAT3, STAT5, and STAT6 as well as antibodies recognizing phosphorylated ALK (p-ALK), JAK2 (p-JAK2), STAT1 (p-STAT1), STAT3 (p-STAT3), STAT5 (p-STAT5), and STAT6 (p-STAT6) were purchased from Cell Signaling Technology (Danvers, MA, USA). The membranes were then incubated with HRP-conjugated secondary antibody (1:1000 dilution, Thermo Fisher Scientific, Waltham, MA, USA) for 1 h at room temperature. Bands were visualized using Pierce ECL Substrate (Thermo Fisher Scientific, Waltham, MA, USA).

Immunoprecipitations were performed on lysates containing 800 μg of total proteins by adding the anti-JAK2 antibody overnight at 4 °C. The immune complexes were precipitated with Protein A-Sepharose 4B (Thermo Fisher Scientific, Waltham, MA, USA) for 2 h at 4 °C. Immunoprecipitated proteins were washed, recovered by boiling in 5 × SDS sample loading buffer, and then separated by SDS-PAGE. Separated proteins were transferred to nitrocellulose membranes at 100 V for 60 min in transfer buffer (20 mM Tris, 150 mM glycine, 20% methanol) and probed with primary antibodies against ALK, JAK2, and p-STAT6.

### Cell Counting Kit-8 (CCK-8) assay

The CCK-8 (Beyotime, Shanghai, China) was used according to the manufacturer’s instructions. Approximately 3 × 10^3^ NIH3T3 or HEK293 cells in the exponential growth phase were seeded into 96-well plates and transiently transfected with pcDNA3.1-EML4-ALK variant 3, control vector, EML4-ALK siRNA, or siRNA-NC for 24, 48, or 72 h. H2228 cells (1 × 10^4^ cells/well) were cultured in 96-well plates for 24 h and then treated with TAE684 (0. 0.001, 0.01, 0.1, 1, 10, or 100 μM) alone or in combination with SH-4–54 (0, 8, 12, 18, 27, 40, or 50 μM) or ruxolitinib (0, 4, 16, 64, 256, 1024, or 4096 μM) for an additional 24 h. Then the CCK8 assay was performed by adding 10 μL of CCK8 solution into each well and incubating for 1 h. The absorbance of each well was measured at 450 nm using a microplate reader (SpectraMax M5, Molecular Devices, CA, USA). All data were calculated from triplicate wells.

### 5-Ethynyl-2’-deoxyuridine (EdU) staining

According to the manufacturer’s instructions, cells were stained with the Cell-Light™ EdU stain kit (RiboBio, Guangzhou, China). Briefly, cells were cultured with 50 μM EdU for 2 h, then washed twice with PBS, and fixed with 4% paraformaldehyde. After permeabilization with 0.5% Triton X-100 and washing with PBS, cells were dyed with Apollo (Red) and Hoechst 33,342 (Blue) for 30 min in the dark and analyzed by fluorescence microscopy.

### Flow cytometry analysis of apoptotic cells

H2228 cells were seeded into 6-well plates (2 × 10^5^ cells/well) and cultured for 24 h. The cells were transfected with siRNA-NC or siRNA-EML4-ALK and cultured for another 24 h. The transfected cells were stained with the Annexin V-FITC Apoptosis Analysis Kit (BD Biosciences, CA, USA) and analyzed by flow cytometry using a FACSAria™ flow cytometer (BD Biosciences, CA, USA).

### Statistical analysis

All data were analyzed using the Statistical Package for Social Sciences (version 16.0., SPSS Inc., Chicago, Illinois, USA). The Student’s t-test identified statistical significance between the two experimental groups. Multivariate analysis of variance identified statistical significance in H2228 cell viability for the three inhibitor groups. *P* < 0. 05 indicated statistical significance.

## Results

### Oncogenic EML4-ALK tyrosine kinase activates JAK-STAT6 signaling pathway

**Fig. 1:**
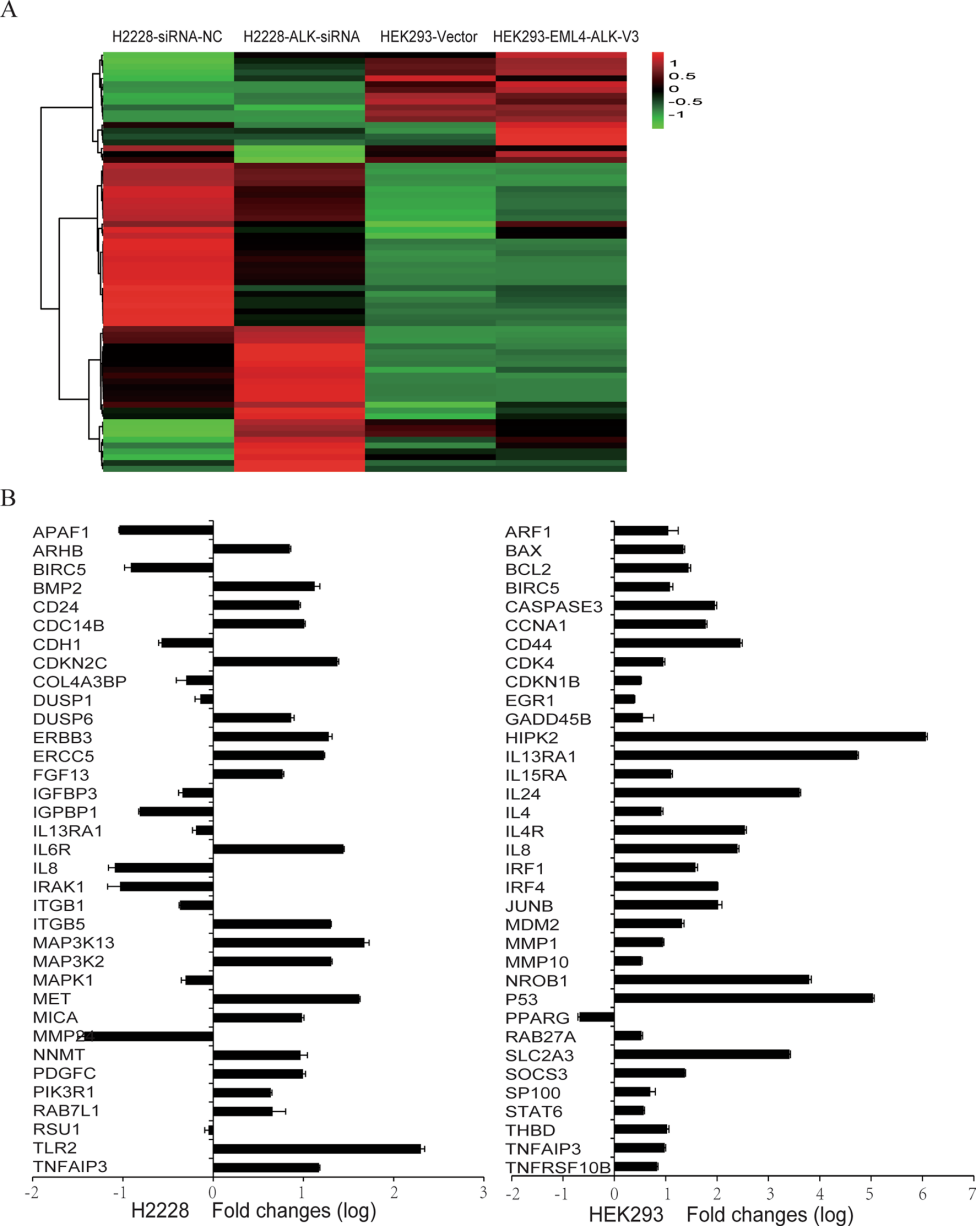
Microarray analysis of H2228 cells without or with EML4-ALK knockdown and of HEK293 cells expressing or not the EML4-ALK variant 3. **a** The heat map shows the regulation of a number of JAK2-STAT pathway genes. **b** Expression of JAK2-STAT pathway-related genes measured by real-time PCR

To explore the mechanisms involved in the transformation of lung cancer cells triggered by EML4-ALK, we used microarray analysis to generate gene expression profiles for H2228 cells transfected with siRNA-NC or siRNA-EML4-ALK. We identified 800 genes upregulated or downregulated, with at least a 1.5-fold change, by EML4-ALK knockdown. Further analysis revealed that many altered genes were involved in the JAK2-STAT pathway (Fig. 1a). Real-time PCR confirmed the microarray results for genes previously reported to be regulated by STAT6 (Fig. 1b).

We also generated differential gene expression profiles for HEK293 cells transfected with EML4-ALK variant 3 or empty vector control. The microarray analysis identified 241 genes upregulated and 21 genes downregulated by EML4-ALK variant 3 (Fig. 1a). Consistent with the EML4-ALK knockdown experiment, the expression of genes involved in the JAK2-STAT pathway, including IL4R, LIF, IL24, IL11, IL15RA, IL6R, SOCS3, and OSMR, was upregulated, which was confirmed by real-time PCR (Fig. 1b). These data indicate that the JAK2-STAT signal pathway may be associated with EML4-ALK function.

### STAT6 is constitutively active in EML4-ALK-positive lung cancer cells and tissues

**Fig. 2:**
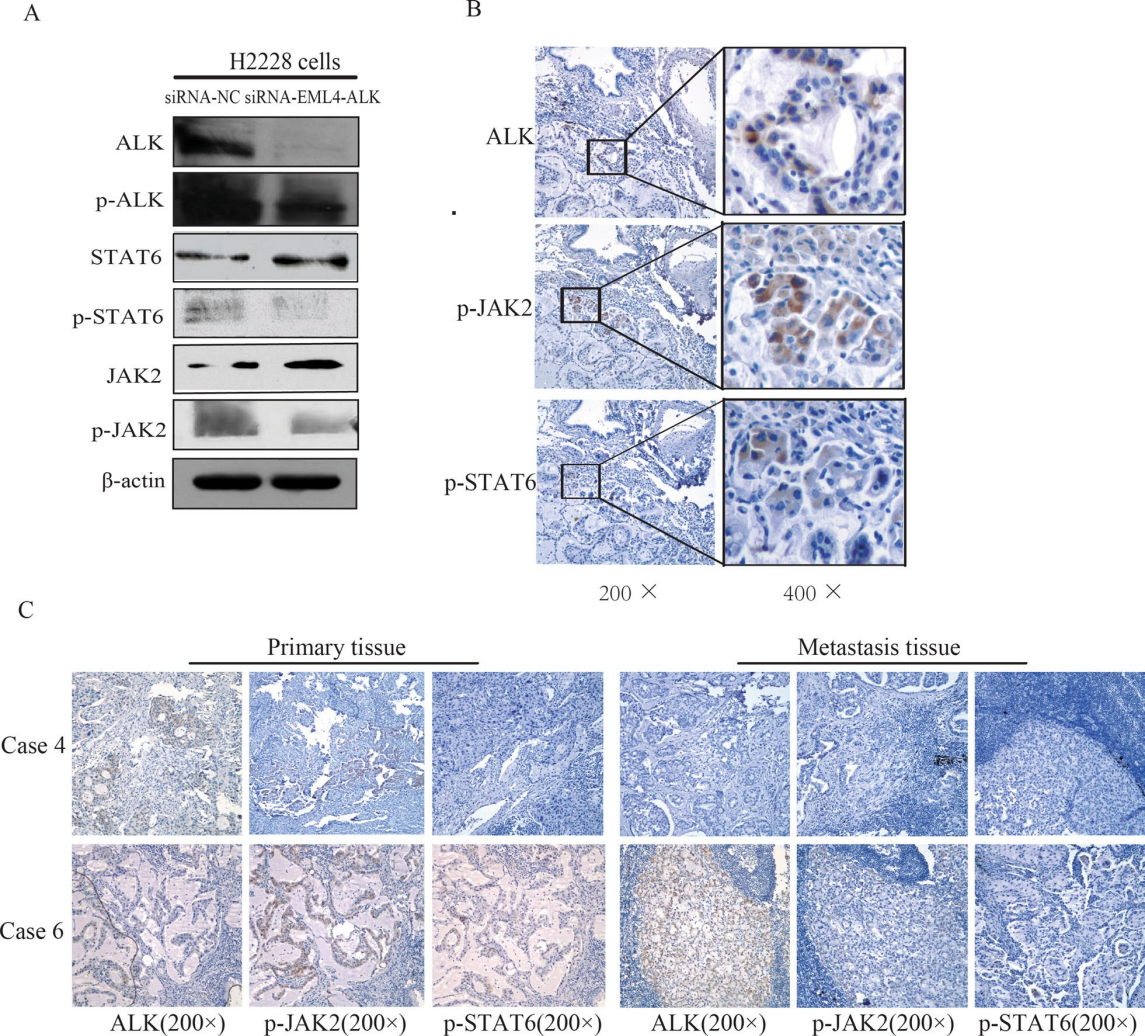
Activation status of STAT proteins in EML4-ALK-positive lung cancer. **a** STAT6 and JAK2 were constitutively phosphorylated in H2228 cells. EML4-ALK knockdown downregulated phosphorylation of JAK2 and STAT6 in H2228 cells. **b** colocalization of ALK, p-STAT6, and p-JAK2 staining in primary tumor tissue. **c** Representative images of ALK,p-JAK2 and p-STAT6 expression by immunohistochemical analysis in the primary tumors and metastatic tumors of EML4-ALK-positive lung cancer patients

To investigate the activation status of STAT proteins in EML4-ALK-positive lung cancer cells, the levels of phosphorylated STATs in H2228 cells were analyzed by immunoblotting. As shown in Fig. 2a, H2228 cells were positive for p-STAT6. JAK2 was constitutively active as p-JAK2 was detected in H2228 cells. Knockdown of EML4-ALK in H2228 cells using siRNA decreased the phosphorylation levels of JAK2 and STAT6.

Immunohistochemistry analyzed the cellular localization of p-STAT6, p-JAK2, and ALK in EML4-ALK-positive lung cancer tissues. As shown in Fig. 2b, p-JAK2 and p-STAT6 appeared in the cytoplasm of lung cancer cells positive for EML4-ALK. As previously reported [10], ALK-positive staining was not seen in the primary tumor cells, indicating the intratumor heterogeneity of ALK rearrangements in primary tumors. Similarly, no p-STAT6 and p-JAK2 staining appeared in the primary tumor cells. It was apparent from comparing the ALK and p-STAT6 positive staining from serial sections that ALK- and p-STAT6-positive areas overlapped (Fig. 2b).

Positive p-STAT6 staining was detected in the primary tumors of 5 out of 7 EML4-ALK-positive lung cancer patients, while p-JAK2 appeared in all seven primary tumors (Table 1, Fig. 2c). Previously, three cases (cases 2-159, 5-177, and 6-184) reportedly showed ALK-positive staining in metastatic tumors [10]. Consistent with the ALK staining, p-JAK2 was present only in the metastasis of these three cases, and p-STAT6 staining did not appear in any metastatic tumors. These data indicated that the JAK/STAT pathway is constitutively active in EML4-ALK-positive lung cancer cells.

### The JAK2-STAT pathway is activated upon EML4-ALK stimulation

**Fig. 3:**
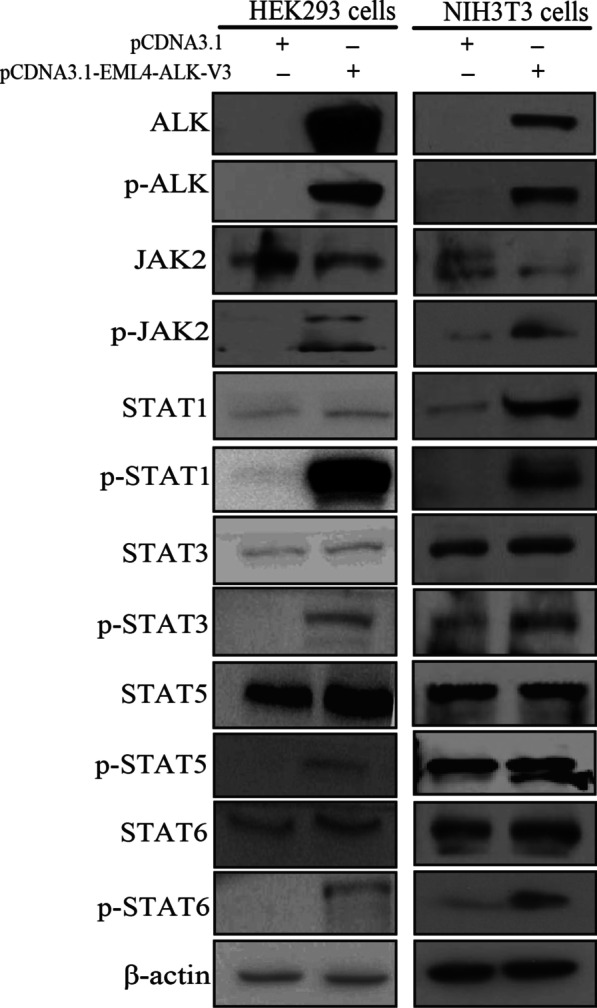
EML4-ALK transfection activated JAK2, STAT1, STAT3, STAT5, and STAT6 in HEK293 and NIH3T3 cells

HEK293 cells, which lack endogenous STAT6 protein but express other components of the IL4 signaling pathway,were used to study the function of EML4-ALK on the JAK2-STAT pathway. We transfected the EML4-ALK variant 3 cDNA plasmid into HEK293 cells. The expression of EML4-ALK variant 3 resulted in the intense activation of JAK2, STAT1, STAT3, STAT5, and STAT6 as demonstrated by an increase of their phosphorylation. Similar results were obtained after EML4-ALK transfection into NIH3T3 cells (Fig. 3). Furthermore, the gene expression profiles indicated that the expression levels of the STAT6 pathway genes (e.g., IL4R, MAF, SOCS3, IL4, IL15RA, and IL6R) were upregulated significantly in EML4-ALK-transfected HEK293 cells compared to those of control HEK293 cells. These data demonstrated that key members of the JAK-STAT signaling pathway might interact with EML4-ALK and be involved in the tumorigenicity mediated by EML4-ALK.

### EML4-ALK interacts with JAK2 and activates the JAK2-STAT pathway by phosphorylation

**Fig. 4:**
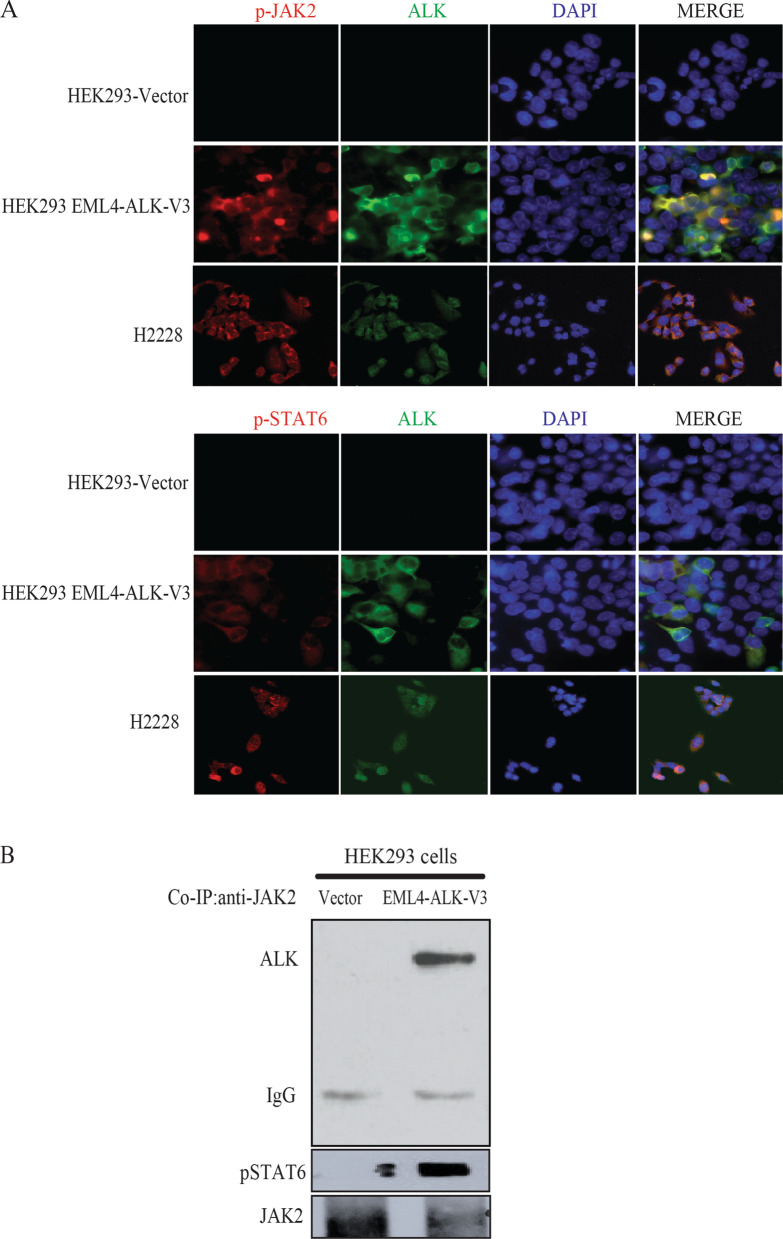
Interactions between components of the JAK2-STAT pathway and EML4-ALK. **a** Active STAT6 and JAK2 colocalized with ALK in EML4-ALK-transfected HEK293 cells and in H2228 cells. **b**: ALK and phosphorylated STAT6 protein were co-immunoprecipitated with JAK2 in EML4-ALK-transfected HEK293 cells

Next, we aimed to characterize the interactions between the JAK2-STAT pathway components and EML4-ALK. Activated STAT6 (p-STAT6) colocalized with ALK in H2228 cells and in HEK293 cells transfected with EML4-ALK (Fig. 4a). We then examined whether EML4-ALK interacted directly with JAK2 to activate the JAK2-STAT pathway. ALK and p-STAT6 co-immunoprecipitated with JAK2 (Fig. 4b) in lysates from EML4-ALK-transfected HEK293 cells, confirming a protein interaction between EML4-ALK and JAK2-STAT6.

### IL4 and IL6 activate JAK2-STAT pathway in EML4-ALK-positive cells

**Fig. 5:**
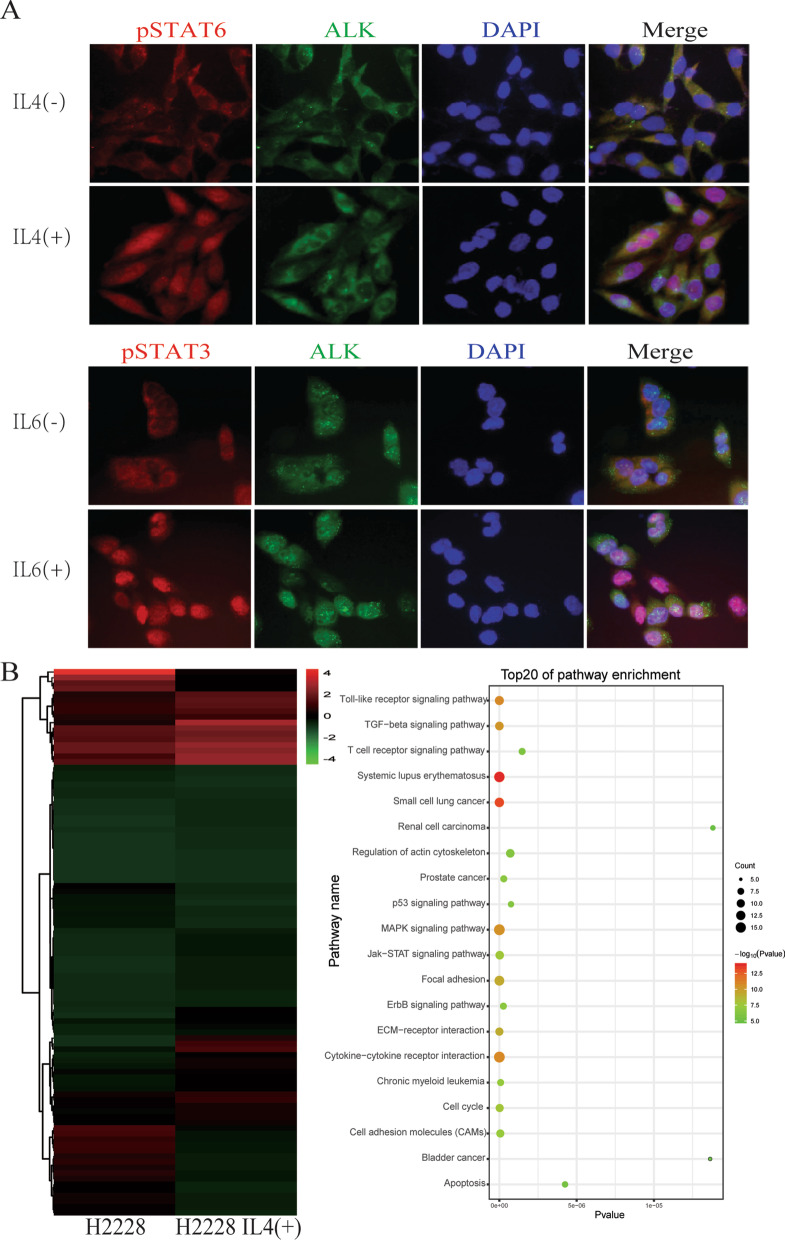
IL4 and IL6 affected the JAK2-STAT pathway in EML4-ALK-positive cells. **a** STAT3 and STAT6 were phosphorylated and translocated to the nuclei of H2228 cells following IL4 or IL6 treatment. **b** Microarray analysis of H2228 cells stimulated or not with IL4. The heat map identifies modified proteins associated with the JAK2-STAT pathway, the regulation of the actin cytoskeleton, the cell cycle, cell adhesion, and the positive regulation of cell proliferation

Because EML4-ALK interacted with the JAK2-STAT pathway resulting in the phosphorylation of STAT proteins in EML4-ALK-positive lung cancer cells, we investigated whether IL4 or IL6 activated the JAK2-STAT pathway in these cells. STAT3 and STAT6 were phosphorylated and translocated into the nucleus of H2228 cells following IL4 or IL6 treatment (Fig. 5a). These data suggested that EML4-ALK was involved in the IL4/IL6/JAK/STAT signaling pathway in EML4-ALK-positive lung cancer cells.

We generated the gene expression profile of H2228 cells unstimulated and stimulated with IL4 by microarray analysis. We identified 280 genes upregulated at least 1.5-fold following IL4 stimulation. Further analyses revealed that several identified genes were involved in the JAK2-STAT pathway, the regulation of the actin cytoskeleton, the cell cycle, cell adhesion, and the stimulation of cell proliferation (Fig. 5b). These data suggested that IL4 activated the JAK2-STAT pathway in EML4-ALK-positive cells.

### Effects of the oncogenic EML4-ALK tyrosine kinase on the biological behaviors of lung cancer cells

**Fig. 6:**
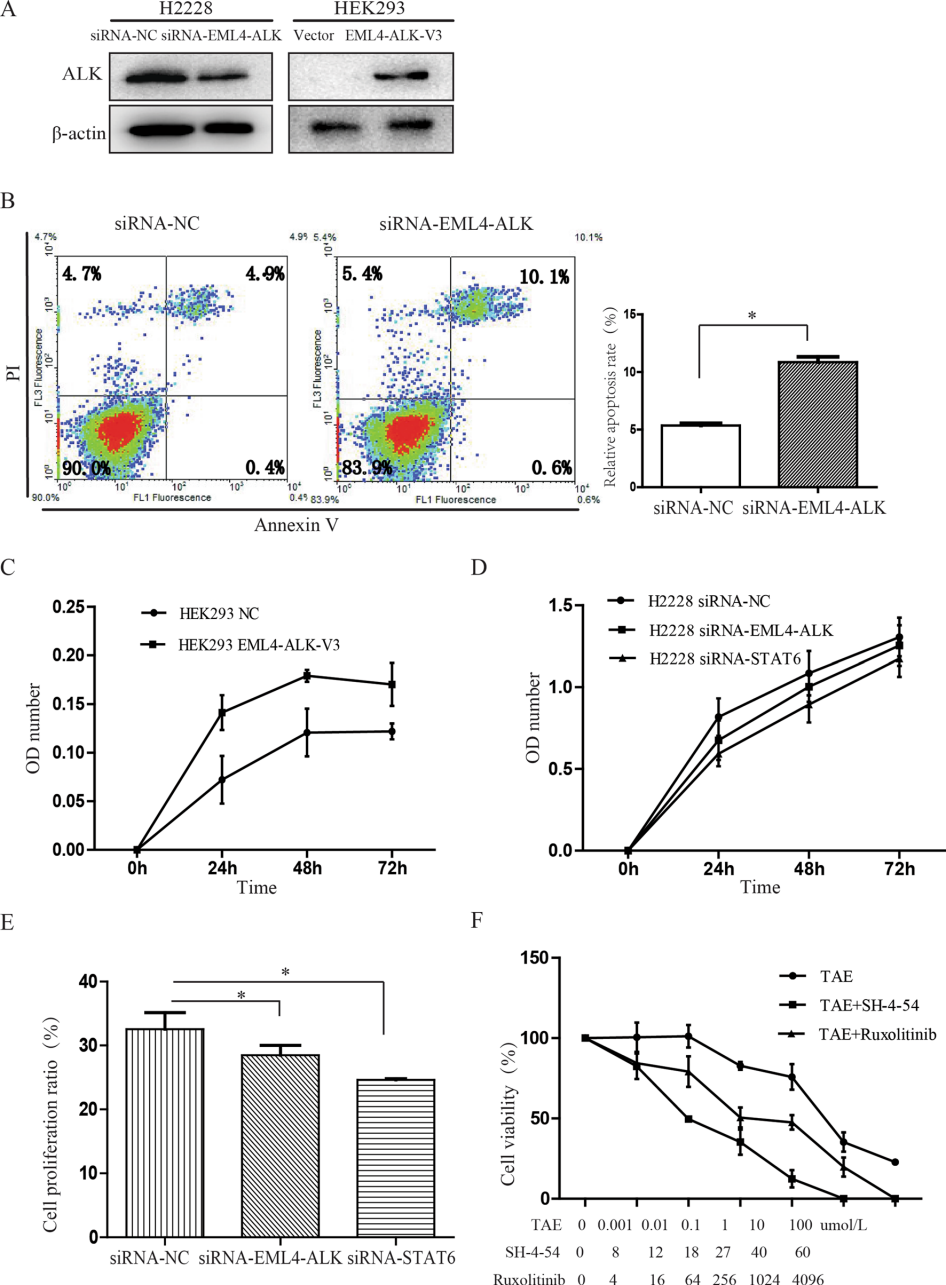
Effect of oncogenic EML4-ALK tyrosine kinase on the biological behaviors of lung cancer cells. **a** EML4-ALK levels in transfected H2228 and HEK293 cells. **b** Increased apoptosis in H2228 cells following EML4-ALK knockdown measured by flow cytometry. **c** Cell viability, measured by the CCK8 assay, was increased by EML4-ALK expression in HEK293 cells. **d**–**e** Viability and proliferation of H2228 cells decreased after knockdown of EML4-ALK or STAT6. **f** H2228 cell viability following treatment with ALK or JAK-STAT pathway inhibitors as measured by the CCK-8 assay. *P < 0.05

To determine the effect of oncogenic EML4-ALK on the biological behaviors of lung cancer cells, we assessed the cell apoptosis following EML4-ALK knockdown in H2228 cells or expression of the EML4-ALK variant 3 in HEK293 cells (Fig. 6a). The percentage of H2228 apoptotic cells increased from 5.36% ± 0.33% to 10.86% ± 079% following EML4-ALK knockdown (*P* = 0.013, Fig. 6b), suggesting an anti-apoptotic role of EML4-ALK.

HEK293 cell viability increased following EML4-ALK expression (*P* < 0.05, Fig. 6c). While exploring EML4-ALK tumorigenicity signaling pathways, we found that the cell viability and DNA replication ability of H2228 cells decreased following knockdown of EML4-ALK or STAT6 using siRNA (Fig. 6d, e). These results indicated that modulation of EML4-ALK or STAT6 levels in EML4-ALK-positive cells might have comparable effects on the biological characteristics of the cells.

Lastly, we analyzed H2228 cell viability after ALK or JAK-STAT pathway inhibitor treatments (Fig. 6f). Compared to the survival of cells treated with ALK inhibitor (TAE684) alone (74.08% ± 12.26%), the viability of EML4-ALK-positive H2228 cells greatly decreased when TAE684 was combined with the JAK2 inhibitor ruxolitinib (54.51% ± 13.64%, *P* = 0.023) or the STAT inhibitor SH-4-54 (39.98% ± 15.02%, *P* = 0.021). These results showed that JAK/STAT signaling acts downstream of EML4-ALK fusion gene to regulate cell proliferation and cell survival in NSCLC cells.

## Discussion

This study suggests that EML4-ALK enhanced the survival of tumor cells and promoted cell proliferation. The silencing of EML4-ALK in H2228 cells, which harbor the EML4-ALK fusion gene, resulted in changes in gene expression from the JAK-STAT signaling, including multiple STAT target genes. The JAK2 and STAT6 phosphorylation levels decreased after EML4-ALK silencing. Moreover, EML4-ALK overexpression stimulated the phosphorylation of JAK2, STAT1, STAT3, and STAT5 in HEK293 cells, suggesting that EML4-ALK activated JAK-STAT signaling. Though HEK293 lacks a functional endogenous STAT6, these results partly confirmed the findings in H2228 cells.

Our results of immunohistochemistry and immunofluorescence assays also showed that STAT6 was continuously phosphorylated and colocalized with ALK in EML4-ALK-positive cells. Co-immunoprecipitation experiments confirmed that JAK2, EML4-ALK, and STAT6 were part of a protein complex and regulated each other. Phosphorylated STAT3 and STAT6 translocated into the nucleus after IL4 and IL6 stimulation of H2228 cells.

Many cytokines, hormones, and growth factors activate the STAT signaling pathway to regulate cell proliferation, differentiation, development, and survival [16]. There are six main STAT family members, STAT1-6. STAT3, STAT5, and STAT6 are strongly activated in breast tumors and act as mammary oncogenes [17]. Constitutive activation of STAT proteins occurs in a variety of blood cancers (e.g., leukemia, lymphoma, and multiple myeloma) [18, 19] and solid tumors (e.g., brain, head and neck, breast, lung, pancreatic, and prostate cancers) [20, 21]. STAT6 activation promotes tumor cell proliferation. Highly expressed STAT6 is an important survival factor for prostate cancer cells and controls the disease progression [22]. Knockdown of STAT3 using siRNA inhibits the growth of tumor cells and induces apoptosis [23]. STAT5 is a key protein in prostate cancer survival as STAT5 gene silencing induces cell death in the prostrate cell lines CWR22RV and LNCaP [24]. In addition, constitutive activation of STAT proteins is closely related to the malignant transformation of cancer and associated with the occurrence and development of tumors [25].

Abnormal activation of the JAK-STAT pathway is a feature of many cancer types [26]. It is associated with tumorigenesis, most likely because the target genes of this pathway encode anti-apoptotic proteins (e.g., B-cell lymphoma 2 [BCL-2] and BCL-X). STAT proteins inhibit tumor cell apoptosis and promote cell proliferation by increasing the levels of anti-apoptotic proteins (BCL-2, BCL-xl. This process induced myeloid leukemia cell differentiation protein [MCL-1]), proliferation-related proteins (e.g., cyclin-D1 and MYC), and the angiogenesis factor vascular endothelial growth factor, and by decreasing p21 and p27 to promote the transition from the G1 to the S phase of the cell cycle. In addition, the coordinated repression of STAT3 and c-Jun restrains FAS-mediated cell apoptosis [27], while the decreased expression of p27kip1 and GRF1-interacting factor 1 (GIF-1) induced by STAT6 controls cell proliferation [28].

In normal cells, STAT activation is usually acute and finely regulated. Indeed, STATs are transported back into the cytoplasm within hours, decreasing the activation signal. JAK-STAT pathway proteins are degraded or deactivated by negative regulation factors (e.g., SOCS and PIAS) in cells [29]. In contrast, constitutively active STATs were found in tumor cells [26]. The continuous STAT activation might result from various mechanisms including [29]: (1) the signaling induced by the action of tumor-secreted, paracrine cytokines, or growth factors, such as IL4 or IL6, on their receptor tyrosine kinase on the cell membrane (e.g., JAK2); (2) the inactivation of negative regulatory factors (e.g., proteases, SOCS, including SOCS-1, phosphatases, PTP1B, PIAS1, and PIAS3); (3) the action of oncogenes and tyrosine kinases (e.g., v-SRC, v-ABL, v-ROS, Etk/BMX, and LCK), which cause sustained activation of STAT3 and STAT6; and (4) JAK point mutations, found in human leukemia cells (e.g., JAK2V617F) and induced the constitutive activation of STAT proteins by JAK2 kinase and abnormal activation of the JAK2-STAT signaling pathway. In patients with primary mediastinal B-cell lymphoma, a point mutation in the STAT6 DNA-binding domain caused the sustained activation of STAT6 [29].

Our results suggest that the JAK2-STAT signaling pathway plays an important role in the occurrence and development of lung cancer mediated by EML4-ALK. Sustained activation of EML4-ALK or ALK caused JAK2 phosphorylation and activation of STAT1, STAT3, STAT5, and STAT6. This study provides the preliminary basis for an interaction mechanism between EML4-ALK and the JAK-STAT pathway. It also brings a new potential role of the JAK-STAT signaling pathway in EML4-ALK-mediated tumorigenesis and biological activity. Although more work is needed to apply our data to the development of therapies, our study provides a novel understanding of targeted lung cancer therapy.

## Conclusions

In conclusion, our data suggest that aberrant expression of EML4-ALK leads to the activation of the JAK2-STAT signaling pathway, which is essential for the development of non-small cell lung cancer.

## Data Availability

All data generated or analysed during this study are included in this published article.The microarray data reported in this paper have been deposited in Gene Expresssion Omnibus(GEO) with the accession code GSE174596 and GSE174772.

## Abbreviations

EML4: echinoderm microtubule-associated protein-like-4
ALK: anaplastic lymphoma kinase
NSCLC: non-small cell lung cancer
JAK2: janus kinase 2
STAT: signal transducer and activator of transcription
EGFR: epidermal growth factor receptor
TKI: tyrosine kinase inhibitors
EdU: 5-ethynyl-2'-deoxyuridine
SOCS: suppressor of cytokine signaling
IL4: interleukin 4
